# Decompressive craniectomy in traumatic brain injury: usage and clinical outcome in a single centre

**DOI:** 10.1007/s00701-017-3418-3

**Published:** 2017-12-12

**Authors:** Teodor Svedung Wettervik, Samuel Lenell, Lena Nyholm, Tim Howells, Anders Lewén, Per Enblad

**Affiliations:** Department of Neuroscience, Section of Neurosurgery, Uppsala University, Uppsala University Hospital, 751 85 Uppsala, Sweden

**Keywords:** Traumatic brain injury, Neurointensive care, Standardised treatment protocol, Decompressive craniectomy, Thiopental

## Abstract

**Background:**

Two randomised controlled trials (RCTs) of decompressive craniectomy (DC) in traumatic brain injury (TBI) have shown poor outcome, but there are considerations of how these protocols relate to real practice. The aims of this study were to evaluate usage and outcome of DC and thiopental in a single centre.

**Method:**

The study included all TBI patients treated at the neurointensive care unit, Akademiska sjukhuset, Uppsala, Sweden, between 2008 and 2014. Of 609 patients aged 16 years or older, 35 treated with DC and 23 treated with thiopental only were studied in particular. Background variables, intracranial pressure (ICP) measures and global outcome were analysed.

**Results:**

Of 35 DC patients, 9 were treated stepwise with thiopental before DC, 9 were treated stepwise with no thiopental before DC and 17 were treated primarily with DC. Six patients received thiopental after DC. For 23 patients, no DC was needed after thiopental. Eighty-eight percent of our DC patients would have qualified for the DECRA study and 38% for the Rescue-ICP trial. Favourable outcome was 44% in patients treated with thiopental before DC, 56% in patients treated with DC without prior thiopental, 29% in patients treated primarily with DC and 52% in patients treated with thiopental with no DC.

**Conclusions:**

The place for DC in TBI management must be evaluated better, and we believe it is important that future RCTs should have clearer and less permissive ICP criteria regarding when thiopental should be followed by DC and DC followed by thiopental.

## Introduction

Traumatic brain injury (TBI) continues to cause substantial morbidity and mortality. The annual incidence is estimated at 260 per 100,000 in Europe, with a fatality rate between 0.9 and 7.6% [14]. There are various treatment protocols for elevated intracranial pressure (ICP), such as ICP-targeted [11] or cerebral perfusion pressure (CPP)-targeted [15], that from different perspectives aim to reduce secondary brain injury. Decompressive craniectomy (DC) was first described in modern neurosurgery, by Kocher [9] and Cushing [4] in the beginning of the twentieth century and has ever since been a matter of much debate. Although life-saving in severe cases, the long-term global outcome of these patients has been shown to be low [18]. In order to more properly evaluate the benefits of DC, two multi-centre randomised controlled trials (RCTs) have recently been conducted [2, 8].

In the DECRA (Decompressive Craniectomy in Patients with Severe Traumatic Brain Injury) study [2], TBI patients with ICP over 20 mmHg continuously or intermittently for 15 min during an hour and who did not respond to first line ICP treatment were randomised to either DC (bifrontotemporoparietal) or continuing standard medical care with addition of mild hypothermia followed by barbiturates. In the Rescue-ICP study [8], patients with ICP above 25 mmHg for 1–12 h, refractory to first- and second-line treatment, were randomised to either barbiturates or DC (unilateral frontotemporoparietal or bifrontal). Neither of the RCTs allowed barbiturates before inclusion. The DECRA study showed better global outcome in the group treated with standard medical care (49% vs 30% favourable outcome). In the Rescue-ICP study, the proportion of patients in good recovery and moderate disability were similar in the treatment groups (approximately 27% favourable outcome).

It is almost impossible to take all possible aspects into consideration when designing RCTs. One risk is that the study protocols applied may be too stereotyped so that the promising treatments evaluated will be disqualified on faulty basis. For example, Kramer et al. [10] showed in a retrospective, observational study that only a fraction of those TBI patients treated with DC were eligible for the RCTs. Indication for DC was in many cases based on clinical and radiological grounds, rather than refractory intracranial hypertension [10]. In the wider patient cohort covered in this observational study, global outcome was favourable in approximately 50% of the DC cases. This finding indicates that DC may have a place in TBI management after all, although overtreatment in less problematic cases cannot be excluded. Under all circumstances, case series studies from single centres are a valuable complement to RCTs. Despite the relatively negative results for DC in the RCTs, we believe that DC may have a role when the treatment protocols for TBI patients are more individualised and the patient selection for DC more refined.

The current study investigates the usage of DC and long-term global outcome in a single centre, both when DC is used as a late step in an escalated management protocol that includes both thiopental as well as DC to reduce refractory elevated ICP, and when DC is done at the time of mass lesion evacuation. The aims were to review the usage of DC and thiopental in the treatment of severe TBI in our centre and to determine if there is a role for DC to achieve favourable outcome.

## Materials and methods

### Patient referral and data collection

The Department of Neurosurgery at the University Hospital in Uppsala, Sweden, provides neurosurgical care for a central part of Sweden, with a population of approximately 2 million people. Most patients are initially managed at local hospitals according to advanced trauma life support (ATLS) principles and then referred to Uppsala (the most distant local hospital 382 km away) [5]. Since 2008, all patients with TBI admitted to our neurointensive care unit are included in the Uppsala Traumatic Brain Injury (TBI) Register [12]. Patients were selected from the Register, which also provided the clinical information required.

### Patients

There were 669 eligible patients ≥16 years, in the Uppsala TBI register between the years 2008–2014. Forty-seven of those patients were excluded due to missing outcome data. The remaining 622 patients were defined as the TBI population. Thirteen of those patients were excluded because of bilateral fixed and dilated pupils on admission (fatal prognosis), leaving 609 patients in the TBI study cohort. The TBI study cohort was divided into four subgroups. (1) *DC group*: 35 patients treated with DC studied in particular. (2) *Thiopental/no DC group*: 23 patients treated with thiopental, but no DC. These patients were also characterised in detail for comparison. (3) *No thiopental/no DC group*: 544 patients, who were neither treated with thiopental nor DC and who did not develop total brain infarction. (4) *Total brain infarction group*: 7 patients not receiving thiopental or DC who developed total brain infarction.

### Neurointensive care

All patients were treated according to the same escalated standardised management protocol summarised below. Treatment goals: ICP ≤20 mmHg, cerebral perfusion pressure (CPP) ≥60 mmHg, systolic blood pressure >100 mmHg, central venous pressure (CVP) 0–5 mmHg, pO_2_ >12 kPa, blood glucose 5–10 mmol/l, electrolytes within normal ranges, normovolemia and body temperature <38 °C. Prophylactic anticonvulsants and muscle relaxants were not given.

#### Step 1

Head elevation 30° in order to facilitate venous outflow and prohibit ventilator-associated pneumonia. Unconscious patients, GCS M 1–5, were intubated, sedated with propofol infusion (Propofol-LipuroB; Braun Medical, Danderyd, Sweden) and received morphine injections or infusions as analgesics. Neurological wake-up tests were frequently performed and sedation was then interrupted. The patients were initially hyperventilated (PaCO_2_, 4.0–4.5 kPa) but normoventilated as soon as ICP was normalised. Extracerebral haematomas and contusions with significant mass effect were surgically evacuated. ICP was monitored in unconscious patients, GCS M 1–5, with either intraventricular drainage catheter or intraparenchymal probes.

If ICP was >20 mmHg, cerebrospinal fluid (CSF) was intermittently drained of small volumes, 1–2 ml, if there was no mass effect. Continuous CSF drainage, was avoided at first, to reduce the risk of not detecting an expanding haematoma and the risk of development of slit ventricles, with incorrect ICP registration. When ICP had been controlled for 1–3 days with intermittent drainage, the ventricular drainage was kept open against a pressure level of 15–20 mmHg.

#### Step 2

If step 1 was inadequate to reduce ICP, step 2 was initiated. Patients were re-evaluated for signs of mass lesions requiring surgery, avoidable factors, insufficient sedation/analgesia or intolerable change to normoventilation. Wake-up tests were not done. To reduce physiological stress response, infusion of 0.2–0.3 mg/kg/24 h β_1_-antagonist Metoprolol (Seloken; AstraZeneca, Södertälje, Sweden) was given and injections of α_2_-agonist Clonidin (Catapresan; BoehingerIngelheim, Stockholm, Sweden) (0.5–1.0 μg/kg × 8 or the same dose as an infusion). Mannitol was only used in case of signs of herniation, as emergency treatment before acute surgery.

#### Step 3

If step 1 and 2 failed to control ICP, thiopental infusion was started (Pentocur; Abcur, Helsingborg, Sweden), provided that no significant mass effect was present. The infusion was initiated with a bolus dose of 4–8 mg/kg given as repeated 50 mg doses until ICP <20 mmHg or blood pressure became unstable. Thereafter, thiopental was continuously infused with 5–10 mg/kg/24 h for 6 h and then 2–5 mg/kg/24 h. The aim was to administer the lowest possible dose to keep ICP <20 mmHg. Burst-suppression on electroencephalogram was not a goal. When thiopental was given, a CPP of 50 mmHg was considered sufficient. Thiopental concentrations >380 μmol/l were avoided.

#### Step 4—Decompressive craniectomy

DC was a last-tier treatment and performed under three conditions: (1) uncontrollable ICP despite thiopental treatment, (2) adverse effects of thiopental and (3) the patient was judged not to tolerate thiopental. The DC of choice was hemicraniectomy if there was a shift of the midline but no significant mass lesions to remove. Bilateral craniectomies with sparing of a bone ridge at the midline were done if no shift was present, usually including the frontotemporoparital regions. The aim was always maximal decompression with removal of as large a bone flap as possible combined with duraplasty.

### Primary decompressive craniectomy

Primary DC (removal of bone flap) in association to evacuation of intracranial haemorrhages was not standard treatment but allowed according to the individual decision by the neurosurgeon.

### ICP analysis

The ICP waveform data were recorded with the Odin software developed at Uppsala University and Edinburgh University [6]. Mean ICP and proportion of good monitoring time (GMT; artefacts and missing data excluded) with ICP >20 mmHg and >25 mmHg, respectively, were calculated during 1-h and 0.5-h periods before and after DC for the DC group. For the thiopental group, mean ICP and proportion of GMT with ICP >20 mmHg and >25 mmHg, respectively, were calculated during 1-h and 0.5-h periods before start of thiopental treatment and after 5 h of infusion, respectively. It was reasonable to believe that thiopental was at appropriate therapeutic level after 5 h loading. The ICP data were also analysed to determine if the patients should have fulfilled the criteria for DECRA (ICP over 20 mmHg continuously or intermittently for 15 min during an hour) and Rescue-ICP (ICP above 25 mmHg for 1–12 h) [2, 8].

### Outcome

Outcome was assessed at 6 months following injury, by specially trained personnel with structured telephone interviews, using the Extended Glasgow Outcome Scale (GOS-E) [17, 20]. The GOS-E contains eight categories of global outcome, from death to upper good recovery. The cut-off for favourable/unfavourable outcome was defined as GOS-E 5–8/1–4.

### Statistical methods

Descriptive statistics were used to present the results and no attempt was made to statistical significance testing, due to the limited number of cases. All nominal data were presented as medians and the interquartile range (Q_1_–Q_3_). Parametric data were presented as the mean ± SD.

## Results

### Place of DC and thiopental in escalated treatment

Table 1 shows the order of the management steps for the 35 patients treated with DC and for the 23 patients treated with thiopental, but no DC. Among the 35 patients in the DC group, 9 patients (26%) were treated stepwise with thiopental before DC, 9 patients (26%) were treated stepwise with no thiopental before DC and 17 patients (49%) were treated with DC as first management step. Six patients (17%) received thiopental after DC (Table 1).

**Table 1:** Patients in various paths of the TBI treatment algorithm

Treatment algorithm^a^	Patients, *n* (%)	Bilateral DC total, *n* (evacuation, *n*)	Hemi-DC total, *n* (evacuation, *n*)	Bone flap total, *n* (evacuation, *n*)
Step 1 → 2 → 3	23 (40)	0 (0)	0 (0)	0 (0)
Step 1 → 2 → 4	9 (16)	1 (1)	6 (3)	2 (2)
Step 1 → 2 → 3 → 4	9 (16)	5 (1)	3 (1)	1 (0)
Step 4	9 (16)	0 (0)	7 (4)	2 (2)
Step 4 at a local hospital^b^	4 (7)	0 (0)	0 (0)	4 (4)
Step 4 → 3	4 (7)	0 (0)	2 (1)	2 (2)
**Total number**	58 (100)	6 (2)	18 (9)	11 (10)

^a^Step 1 = Basal treatment, Step 2 = No wake-up tests and stress relief medication, Step 3 = Thiopental, Step 4 = DC

^b^Two patients received thiopental after DC

### DC group

The 35 patients treated with DC are characterised in Table 2. The DC group had a mean age of 40 years and 83% were male. On admission, 15% were in GCS M 1–2, 43% showed pupil abnormality and 38% had CT Marshall score III-IV). Hemi-DC was performed in 18 patients (in association with haematoma evacuation in 9 cases), bilateral DC in 6 (in association with haematoma evacuation in 2 cases) and the bone flap was removed in 11 patients (in association to evacuation of intracranial haemorrhages in 10 and after previous evacuation in one) (Table 1). In association with the DC, the haematoma evacuation was extracerebral in 13 patients, intracerebral in 5 and both in 3.

**Table 2:** Background variables of the subgroups of the TBI study cohort

Background^a^	DC	Thiopental/no DC	No thiopental/no DC	Total brain infarction, no thiopental/no DC
Total, *n*	35	23	544	7
Mean age (years)	40	37	51	61
Male, *n* (%)	29 (83)	15 (65)	423 (78)	7 (100)
GCS M at admission				
1–2, *n* (%)	5 (15)	4 (17)	20 (4)	5 (71)
3–6, *n* (%)	29 (85)	19 (83)	524 (96)	2 (29)
Pupil abnormality, *n* (%)	15 (43)	5 (23)	70 (13)	6 (86)
CT Marshall score				
DI I, *n* (%)	0 (0)	1 (5)	10 (2)	0 (0)
DI II, *n* (%)	6 (18)	6 (27)	283 (52)	1 (14)
DI III, *n* (%)	11 (32)	8 (36)	67 (12)	1 (14)
DI IV, *n* (%)	2 (6)	1 (5)	24 (4)	1 (14)
Evacuated V, *n* (%)	15 (44)	6 (27)	107 (17)	1 (14)
Non-evacuated VI, *n* (%)	0 (0)	0 (0)	52 (10)	3 (43)
Median score (IQR)	4 (3–5)	3 (2–5)	2 (2–5)	5 (3–6)

^a^Missing data: one DC patient GCS M score, one DC patient CT Marshall score, one thiopental/no DC patient pupil abnormality, one thiopental/no DC patient CT Marshall score, one no thiopental/no DC patient pupil abnormality and one no thiopental/no DC patient CT Marshall score

Among the nine patients treated stepwise with thiopental before DC, a bilateral DC was performed in five patients (concurrent evacuation intracranial haemorrhages in one), hemi-DC in three (concurrent evacuation in one) and the bone flap was removed in one patient after earlier evacuation (Table 1). In the nine patients treated stepwise with no thiopental before DC, a bilateral DC was performed in one patient simultaneously with evacuation of haematoma, hemi-DC in six (concurrent evacuation in three) and the bone flap was not put back in two patients after haematoma evacuation (Table 1). Among the 17 patients treated with DC as first management step, none had bilateral DC, nine had hemi-DC (concurrent evacuation in five) and in eight the bone flap was not put back after haematoma evacuation (life-saving operation at local hospitals in four) (Table 1).

Significant mass effect, defined as midline shift ≥5 mm, was present before surgery in 13/16 (81%) of the hemi-DC cases and in none of the 6 bilateral DC cases (Table 3). Compressed/absent basal cisterns were seen in 8/16 (50%) of the hemi DC cases and in 6/6 (100%) of the bilateral DC cases (Table 3). Among the patients in whom the bone flap was not put back, the midline shift was ≥5 mm in 10/11 (91%) and compressed/absent basal cisterns in 7/11 (64%) (Table 3).

**Table 3:** CT findings before DC or thiopental

CT before treatment^a^	DC all	Hemi- DC	Bilateral DC	Bone flap	Thiopental/no DC
Midline shift ≥5 mm, *n* (%)	23 (70)	13 (81)	0 (0)	10 (91)	1 (4)
Compressed/absent basal cisterns, *n* (%)	21 (64)	8 (50)	6 (100)	7 (64)	3 (13)

^a^Missing data: two hemi-DC patients

ICP data for 16 patients with ICP monitor prior to DC (ICP data unrecorded in one bilateral DC and one bone flap DC case) are presented in Table 4. In the hemi DC group, mean ICP during 0.5 h prior to DC was 27.5 mmHg and 8.8 mmHg during 0.5 h after and the proportion of GMT >25 mmHg was 56% before and 0% after for the same periods. For the bilateral DC group, mean ICP during 0.5 h prior to DC was 23.4 mmHg and 11.9 mmHg during 0.5 h after and the proportion of GMT >25 mmHg was 39% before and 18% after. Eight out of nine (89%) fulfilled the inclusion criteria for DECRA in the hemi-DC group and 5/5 (100%) in the bilateral DC group. The inclusion criteria for Rescue-ICP were fulfilled in 5/9 (56%) in the hemi-DC group and in none of the patients in the bilateral DC group (Table 4).

**Table 4:** ICP before and after DC and thiopental, respectively, and the proportion of patients fulfilling the inclusion criteria for the DECRA and Rescue-ICP RCTs

ICP measure^a^	Hemi-DC	Bilateral DC	Bone flap	All DC	ICP measure^a^	Thiopental/no DC
Mean ICP 1 h before DC (mm Hg)	25.1 (±8.8)	23.8 (±2.7)	17.1 (±9.7)	23.4 (±7.4)	Mean ICP 1 h before thiopental (mm Hg)	18.8 (±7.0)
Mean ICP 1 h after DC (mm Hg)	10.2 (±6.5)	11.1 (±12.3)	10.6 (±3.9)	10.6 (±7.1)	Mean ICP hour 5–6 after thiopental (mm Hg)	17.1 (±5.7)
Mean ICP 0.5 h before DC (mm Hg)	27.5 (±13.5)	23.4 (±5.7)	16.7 (±10.2)	24.3 (±10.9)	Mean ICP 0.5 h before thiopental (mm Hg)	20.2 (±7.9)
Mean ICP 0.5 h after DC (mmHg)	8.8 (±6.5)	11.9 (±13.4)	8.3 (±4.0)	9.4 (±7.6)	Mean ICP hour 5–5.5 after thiopental (mm Hg)	17.0 (±5.5)
GMT >20 mmHg 1 h before DC (%)	67 (±41)	75 (±19)	39 (±55)	65 (±37)	GMT >20 mmHg 1 h before thiopental (%)	46 (±30)
GMT >20 mmHg 1 h after DC (%)	2 (±6)	20 (±44)	8 (±12)	7.5 (±22)	GMT >20 mmHg hour 5–6 after thiopental (%)	37 (±37)
GMT >20 mmHg 0.5 h before DC (%)	70 (±48)	67 (±27)	26 (±37)	62 (±41)	GMT >20 mmHg 0.5 h before thiopental (%)	57 (±35)
GMT >20 mmHg 0.5 h after DC (%)	0 (±1)	20 (±42)	8 (±17)	7 (±22)	GMT >20 mmHg hour 5–5.5 after thiopental (%)	36 (±41)
GMT >25 mmHg 1 h before DC (%)	54 (41)	41 (10)	15 (±21)	45 (±34)	GMT >25 mmHg 1 h before thiopental (%)	21 (±26)
GMT >25 mmHg 1 h after DC (%)	0 (±0)	19 (±43)	1 (±2)	5 (±20)	GMT > 25 mmHg hour 5–6 after thiopental (%)	12 (±23)
GMT >25 mmHg 0.5 h before DC (%)	56 (±42)	39 (±37)	18 (±25)	45 (±38)	GMT > 25 mmHg 0.5 h before DC (%)	28 (±32)
GMT >25 mmHg 0.5 h after DC (%)	0 (±0)	18 (±41)	2 (±4)	5 (±20)	GMT > 25 mmHg hour 5–5.5 after thiopental (%)	10 (±22)
DECRA qualification, *n* (%)	8 (89)	5 (100)	1 (50)	14 (88)		17 (89)
Rescue-ICP qualification, *n* (%)	5 (56)	0 (0)	1 (50)	6 (38)		3 (16)

^a^Mean ICP (mm Hg) and proportion of good monitoring time (GMT) with ICP >20 mmHg and >25 mmHg, respectively, during 1-h and 0.5-h periods before and after DC. Mean ICP and proportion of good monitoring time (GMT) with ICP >20 mmHg and >25 mmHg, respectively, during 1-h and 0.5-h periods before start of thiopental treatment and after 5 h from start in the thiopental group. Missing ICP data: Bilateral DC = 1, Bone flap = 1, Thiopental/no DC = 4

Favourable outcome (GOS-E 5–8) was seen in 14/35 (40%) of all DC patients and 6/35 (17%) of the DC patients died (Table 5). Hemi-DC, bilateral DC and removal of bone flap had 22% (4/18), 67% (4/6) and 55% (6/11) favourable outcome, respectively, and 17% (3/18), 0% (0/6) and 27% (3/11) mortality, respectively (Table 6).

**Table 5:** GOS-E at 6 months for the subgroups of the TBI study cohort

GOS-E at 6 months	DC	Thiopental/no DC	No thiopental/no DC	Total brain infarction, no thiopental/no DC
1 (dead total), *n* (%)^a^	6 (17)	1 (4)	62 (11)	7 (100)
1a (dead at the NIC-U), *n* (%)^b^	3 (9)	1 (4)	17 (3)	7 (100)
2 (vegetative), *n* (%)	3 (9)	2 (9)	4 (1)	0 (0)
3 (lower severe), *n* (%)	12 (34)	8 (35)	83 (15)	0 (0)
4 (upper severe), *n* (%)	0 (0)	0 (0)	45 (7)	0 (0)
5 (lower moderate), *n* (%)	6 (17)	0 (0)	40 (8)	0 (0)
6 (upper moderate), *n* (%)	3 (9)	2 (9)	61 (11)	0 (0)
7 (lower good), *n* (%)	2 (6)	1 (4)	98 (18)	0 (0)
8 (upper good), *n* (%)	3 (9)	9 (39)	151 (28)	0 (0)
Total *n*	35	23	544	7
Median GOS-E grade (IQR)	3 (2–5)	6 (3–8)	6 (3–8)	1 (1–1)
Favourable (GOS-E 5–8), *n* (%)	14 (40)	12 (52)	350 (64)	0 (0)

^a^All patients dying

^b^Patients dying at the neurointensive care unit (NIC-U)

**Table 6:** Type of DC versus GOS-E at 6 months

Type of DC	Hemi-DC	Bilateral DC	Bone flap
Total, *n* (%)	18 (51)	6 (17)	11 (31)
Mortality, *n* (%)	3 (17)	0 (0)	3 (27)
Median GOS-E grade (IQR)	3 (2–3)	5 (2–5)	5 (1–7)
Favourable (GOS-E 5–8), *n* (%)	4 (22)	4 (67)	6 (55)

Favourable outcome was seen in 4/9 (44%) of the patients 293 treated stepwise with thiopental before DC, in 5/9 (56%) of the patients treated stepwise with no thiopental before DC and in 5/17 (29%) among the patients treated with DC as first management step (Table 7).

**Table 7:** Outcome for nine patients treated stepwise with thiopental before DC, nine patients treated stepwise with no thiopental before DC and 17 patients treated with DC as first management step

GOS-E at 6 months	Step 1 → 2 → 3 → 4^a^	Step 1 → 2 → 4	Step 4^b^
1 (dead), *n* (%)	1 (11)	1 (11)	4 (24)
2 (vegetative), *n* (%)	2 (22)	0 (0)	1 (6)
3 (lower severe), *n* (%)	2 (22)	3 (33)	7 (41)
4 (upper severe), *n* (%)	0 (0)	0 (0)	0 (0)
5 (lower moderate), *n* (%)	0 (0)	3 (33)	3 (18)
6 (upper moderate), *n* (%)	2 (22)	1 (11)	0 (0)
7 (lower good), *n* (%)	0 (0)	0 (0)	2 (12)
8 (upper good), *n* (%)	2 (22)	1 (11)	0 (0)
Total number	9	9	17
Median grade (IQR)	3 (2–7)	5 (3–6)	3 (1–5)
Favourable (GOS-E 5–8), *n* (%)	4 (44)	5 (56)	5 (29)

^a^Step 1 = Basal treatment, Step 2 = No wake-up tests and stress relief medication, Step 3 = Thiopental, Step 4 = DC

^b^Six patients later received thiopental after DC

### Thiopental, no DC group

The characteristics of the 23 patients treated with thiopental and no DC are presented in Table 2. All patients were treated stepwise (step 1 → 3) according to the TBI treatment protocol (Table 1). The mean age was 37 and 65% were male, which was similar to the DC group (Table 2). On admission 4/23 (17%) were in GCS M 1–2, 5/22 (23%) showed pupil abnormality and 9/22 (41%) had Marshall CT score III-IV. Compared to the DC group these patents were less likely to have pupil abnormalities (Table 2). Midline shift ≥5 mm was present in 1/23 (4%) and basal cisterns were compressed/absent in 3/23 (13%) in this group (Table 3).

ICP data for this group are presented in Table 4. Mean ICP during 0.5 h before start of thiopental was 20.2 mmHg and 17.0 mmHg during 5–5.5 h after start and the proportion of GMT >25 mmHg was 28% before and 10% after start for the same periods. The inclusion criteria for DECRA were fulfilled in 17/19 (89%) of the patients for Rescue-ICP in 3/19 (16%) of the patients (Table 4).

Favourable outcome (GOS-E 5–8) was seen in 12/23 (52%) of the patients treated with thiopental without DC and 1/23 (4%) died (Table 5).

### No thiopental/no DC group

The 544 patients who did not receive thiopental and did not undergo DC (no DC/no thiopental group) had a mean age of 51 years and 78% were male (Table 2). The admission status was better than for either the Thiopental or the DC groups, with 20/544 (4%) in GCS M 1–2, pupil abnormality in 70/543 (13%) and Marshall CT score III-IV in 91/543 (17%) of the cases (Table 2). The patients were treated stepwise according to the TBI treatment protocol, but, by definition, no patient went to step 3 or 4.

Favourable outcome (GOS-E 5–8) was seen in 350/544 (64%) of the patients who neither received thiopental nor was operated on with DC and 62/544 (11%) died (Table 4).

### No thiopental/no DC—total brain infarction (TBI study cohort)

The seven patients in the TBI study cohort who developed total brain infarction and were neither treated with thiopental nor DC had a mean age of 61 years and all were male (Table 2). The admission status was poor, with 5/7 (71%) in GCS M 1–2, pupil abnormality in 6/7 (86%) and Marshall score VI [non-evacuated mass lesion in 3/7 (43%)].

## Discussion

According to our standardised escalated TBI management protocol, DC was a late step to control ICP. Primary DC (removal of bone flap) in association to evacuation of intracranial haemorrhages was not standard treatment but allowed in selected cases decided by the responsible neurosurgeon. The results showed that DC was performed in 18/35 (51%) as a stepwise procedure and in 17/35 (49%) as a primary procedure (Table 1).

Looking at the 18 cases treated with DC in an escalated manner, DC was done after thiopental in nine cases and without thiopental in nine cases. A bilateral DC was done in five of the nine cases receiving thiopental preoperatively and in one of the nine cases not receiving thiopental. Among all 18 hemi-DC cases, midline shift ≥5 mm was present in 81% (13/16) and in none of all six bilateral DC cases. In the thiopental/no DC group, midline shift ≥5 mm was only seen in one out of 23 patients (4%). These results reflect our philosophy that if there is no mass effect present, thiopental comes before DC, while if there is a significant mass effect thiopental is not an option and instead a hemi-DC should be done, and if there are ICP problems and no midline shift a bilateral DC is preferred.

Notable in this study is that in a substantial number of cases treated with thiopental, DC was never required (thiopental/no DC group) and 52% of those 23 patients had favourable outcome and only one patient died. It is also important to note that in 28% of the cases that received thiopental in a stepwise manner a DC was needed. These findings strongly indicate, firstly, that thiopental should be used before DC (provided that there is no mass effect), taking into consideration also all efforts required and all problems involved with the replacement of the bone later [21], and secondly, that it is important that DC is performed promptly if thiopental is insufficient when this principle is applied. It is difficult to know whether the latter was the case in the two RCTs [2, 8]. In the DECRA study, lifesaving DC was allowed after a period of 72 h had elapsed since admission and ICP >20 mmHg for 4 h or >30 mmHg for 1 h in the medical group [2]. In the Rescue-ICP study, it was stated that if the patient subsequently deteriorated (for example, prolonged and unacceptably high ICP >40 mmHg with compromised CPP) a DC was allowed [8]. With those study designs, it cannot be excluded that the question regarding either DC or thiopental was evaluated rather than the order of those treatments. Hence, we believe that to evaluate the place for DC in the management of TBI better, future RCTs should have clearer and less permissive ICP criteria for when DC should be performed in patients receiving thiopental and when thiopental should be given to patients operated with DC, respectively.

Looking at the ICP data (Table 4), it is obvious that there were significant ICP problems before DC as well as before start of thiopental (indicating that the patients were not over-treated) and that both treatments had substantial effect on ICP in the situations when applied. It is also apparent that different summary measures of ICP give a different impression of the magnitude of the ICP problems, which must be considered when designing RCTs. For example, patients showing pronounced repeated plateau waves may show a relatively low mean ICP, while a substantial proportion of GMT will have ICP >20 or >25 mm of Hg. In this study, 89%, 100% and 89% of the patients treated with hemi-DC, bilateral DC and thiopental without DC, respectively, would have qualified for DECRA, but a much smaller proportion fulfilled the criteria for the Rescue-ICP where the ICP requirements were much higher.

**Fig. 1:**
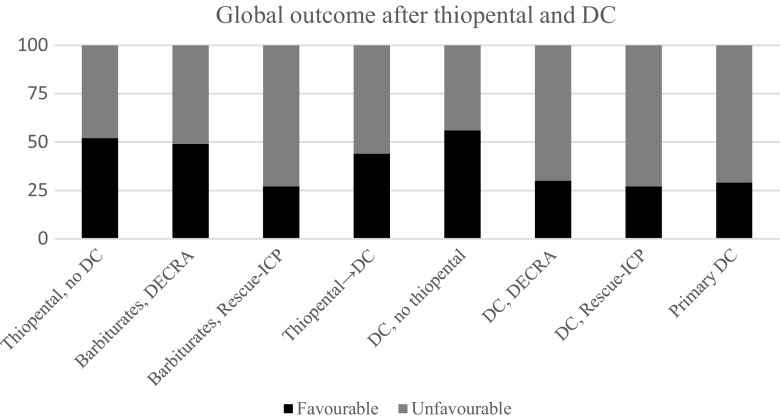
**Clinical outcome after thiopental and DC.** The bars named with DECRA or Rescue-ICP were based on data from those studies [2, 8]. The other bars were from the study groups of this article. Favourable outcome was defined as GOS-E 5–8 and unfavourable outcome as GOS-E 1–4

Favourable outcome was seen in 4/9 (44%) of the patients treated stepwise with thiopental before DC, in the 5/9 (56%) of the patients treated stepwise with no thiopental before DC and in 5/17 (29%) among the patients treated with DC as first management step. Considering that there were substantial ICP problems and that DC was either a late treatment step or judged to be necessary early due to severe injuries with brain swelling (preoperative and/or perioperative massive brain swelling without significant mass lesions in all cases, data not presented), those results appear relatively good overall. It is, however, difficult to compare the results with other studies due to differences in patient characteristics and management algorithms, but if not considering those differences, other non-randomised studies have reported favourable outcome in, for example, 69% [19], 71% [13] and 68% [16]. The reason why the DECRA and Rescue-ICP studies report less favourable results may of course be explained by inclusion of more severe cases but also possibly by less individualised management. Figure 1 shows clinical outcome for the patients in our study in comparison to these two RCTs.

One of the aims of this study was to evaluate the usage of DC, and we found that DC was not only used as a late option in the escalated management protocol but also during emergency procedure in association with evacuation of intracranial haemorrhage, which was decided by the individual neurosurgeon. This observation is in accordance with an earlier study of the management of post-traumatic mass lesions showing that DC was practiced in association with one-third of the acute emergency evacuations [1]. This practice of primary DC is not really studied in the DECRA and Rescue-ICP studies. Specific studies of early DC associated with haematoma evacuation have been asked for by Coplin et al. [3] and one attempt is the ongoing Rescue-ASDH study [7].

When the management is escalated according to a stepwise protocol, it is important to evaluate whether the patient has potential to survive with reasonable quality of life. Looking at the patients who were treated neither with thiopental nor DC and developed total brain infarction, it was obvious that they were deemed to have a very dismal prognosis according to age, neurological grade and CT findings.

The results of this study must be interpreted with caution due to the limited number of cases included. This fact and that the treatment followed a staged management protocol also makes it difficult to compare different management strategies and to define characteristics favouring certain treatments or prognostic factors. However, the results reflect in a descriptive way how DC is used in real practice in a much more complex situation than was evaluated in the recent RCTs. We hope that this paper will contribute to keeping the discussion alive regarding the place of DC (and also thiopental) and to stimulate the initiation of further studies, despite the relatively poor results reported by the RCTs.

## Conclusions

DC was used in compliance with the escalated local standardised management protocol and to some extent as a primary management procedure in association with evacuation of mass lesion. Thiopental was used before DC if there was no mass effect present. It was apparent that thiopental was sufficient in many cases but also needed to be followed by DC in many cases. Unilateral DC was performed if there was midline shift and bilateral if not. Analysis of preoperative ICP showed that there was a clear indication for both thiopental and DC. The proportion of favourable outcome appeared acceptable with favourable outcome in 44% of the patients treated stepwise with thiopental before DC, in the 56% of the patients treated stepwise with no thiopental before DC and in 29% among the patients treated with DC as first management step. More studies are required to evaluate the place for DC in the management of TBI better and we believe it is important that future RCTs should have clearer ICP criteria that are less permissive for when thiopental should be followed by DC and DC followed by thiopental, respectively.
